# Archaeal membrane-associated proteases: insights on *Haloferax volcanii* and other haloarchaea

**DOI:** 10.3389/fmicb.2015.00039

**Published:** 2015-02-06

**Authors:** María I. Giménez, Micaela Cerletti, Rosana E. De Castro

**Affiliations:** Instituto de Investigaciones Biológicas, Facultad de Ciencias Exactas y Naturales, Universidad Nacional de Mar del Plata, Consejo Nacional de Investigaciones Científicas y TécnicasMar del Plata, Argentina

**Keywords:** archaeal proteolysis, membrane-associated proteases, *Haloferax volcanii*, cell envelope, S-layer glycoprotein

## Abstract

The function of membrane proteases range from general house-keeping to regulation of cellular processes. Although the biological role of these enzymes in archaea is poorly understood, some of them are implicated in the biogenesis of the archaeal cell envelope and surface structures. The membrane-bound ATP-dependent Lon protease is essential for cell viability and affects membrane carotenoid content in *Haloferax volcanii*. At least two different proteases are needed in this archaeon to accomplish the posttranslational modifications of the S-layer glycoprotein. The rhomboid protease RhoII is involved in the *N*-glycosylation of the S-layer protein with a sulfoquinovose-containing oligosaccharide while archaeosortase ArtA mediates the proteolytic processing coupled-lipid modification of this glycoprotein facilitating its attachment to the archaeal cell surface. Interestingly, two different signal peptidase I homologs exist in *H. volcanii*, Sec11a and Sec11b, which likely play distinct physiological roles. Type IV prepilin peptidase PibD processes flagellin/pilin precursors, being essential for the biogenesis and function of the archaellum and other cell surface structures in *H. volcanii.*

## INTRODUCTION

Membrane-associated proteases participate in a variety of processes essential for cell physiology including membrane protein quality control, processing of exported and/or membrane-anchored polypeptides, regulatory circuits, cell-signaling, the stress response and assembly of cell surface structures (Akiyama, 2009; Dalbey et al., 2011; Schneewind and Missiakas, 2012; Konovalova et al., 2014). Their targets are mainly membrane-bound or secreted proteins which account for 20–30% of total proteins encoded in most genomes (Wallin and von Heijne, 1998) and include membrane receptors, structural proteins, transporters and enzymes such as transferases, oxidoreductases, and hydrolases.

Integral membrane proteases comprise two distinct groups. The first group is represented by peptidases anchored to the cytoplasmic membrane that exert their catalytic activity in an aqueous compartment (cytoplasm, periplasm, or extracellular milieu) either at the aqueous-membrane boundary or after the substrate has been released or extracted from the membrane. Within this category are signal peptidases (SP), site 1 proteases (S1P) or sheddases, signal peptide hydrolases SPPA, HtpX, sortases and the energy-dependent proteases FtsH and LonB. The second group is represented by the so-called intramembrane cleaving proteases (ICliPs) which have their active sites immersed in the hydrophobic environment of the membrane (Wolfe, 2009; Dalbey et al., 2012). This group includes GxGD-aspartyl proteases (eukaryal signal peptide peptidase SPP and presenilin families), rhomboids and site 2 proteases (S2P).

*Archaea*, one of the three domains of life, are widespread in nature but predominate in environments with extreme values of pH, temperature, salt concentration and pressure (Robertson et al., 2005). Studies on archaeal biology are encouraged as they provide the opportunity to better understand cell physiology as well as extend the resources for biotechnology.

The genome sequences of archaea show that these unusual organisms encode a variety of proteolytic enzymes some of which have been characterized (Ward et al., 2002; Maupin-Furlow et al., 2005; De Castro et al., 2006; Ng et al., 2007). Most of the membrane protease families found in bacteria and/or eukaryotic cells also occur in archaea, however, the role of these enzymes in the context of the archaeal cell is poorly understood. In the last decade a number of studies have started to advance the knowledge on this field (see references in **Table 1**). This mini review describes what is known about proteases associated with the cell surface of archaeal cells on the basis of complete genome sequences and biochemical and/or genetic studies. Emphasis will be placed on the proteolytic enzymes affecting the cell envelope and surface structures of the euryarchaeon *Haloferax volcanii* and other haloarchaea. *H. volcanii* grows in a wide range of salinity (1.5–3.5 M NaCl) and is a model organism to study archaeal biology due to a number of advantages including the simplicity of its culture conditions, availability of complete genome sequences and feasibility of its genetic manipulation.

**Table 1:** Predicted membrane proteases of represtenative archaeal genomes.

Protease name	MEROPS family	Cleavage site	Topology	Description	Organism	Reference
**Signal peptidases (SP)**				Cleaves signal peptides of preproteins		
**SPI**	S26B	Active site on external face of cytoplasmic membrane		Resemble eukaryotic SPI. Cleaves the majority of signal peptides from preproteins exported by Sec (Tat?) system		
SPI (MJ0260)			2 TMS	First SPI characterized from archaea	*Methanococcus voltae*	Ng and Jarrell (2003); Ng et al. (2007)
Sec11a (HVO_2603); Sec11b (HVO_0002)			1 TMS	Both active *in vitro*, different efficiency for substrate cleavage. Sec11b is essential for viability	*Haloferax volcanii*	Fine et al. (2006); Fink-Lavi and Eichler (2008)
**TFPP-like (SPIII)**		Active site on internal face of cytoplasmic membrane		Novel family of aspartyl proteases (GxGD). Cleaves signal peptides of preflagellins prior to incorporation into the archaellum		
**PibD** (SSO0131)	A24		5 TMS	Broad substrate range. Cleaves signal peptides of preflagellins and sugar-binding proteins (“bindosome”)	*Sulfolobus solfataricus*	Albers et al. (2003, 2006); Szabo et al. (2006); Ng et al. (2009)
(Saci_0139)			6 TMS	*In vivo* activity assays led to redefine PibD as GxHyD (Hy = hydrophobic residue) rather than a GxGD group of proteases	*Sulfolobus acidocaldarius*	Henche et al. (2014)
(HVO_2993)			6 TMS	Cleaves preflagellins and type IV pilin-like proteins. Required for swimming motility (flagella) and surface adhesion (type IV pili-like structures)	*H. volcanii*	Tripepi et al. (2010)
**EppA** (MMP0232, MJ0835.2)	A24A		9–10 TMS	Cleaves signal peptides of pilin-like proteins containing a DUF361 domain	*Methanococcus maripaludis*, *Methanococcus jannaschii*	Szabo et al. (2007)
**FlaK** (Mvol_0164)	A24B		5 TMS	Inactivation of FlaK generates non- flagellated cells. Residues Ser52, His122 and Asp148 crucial for protease activity	*M. voltae*	Bardy and Jarrell (2003); Bardy et al. (2005)
(MMP0555)			5 TMS	Displays preflagellin peptidase activity. placeCityCrystal structure is available	*M. maripaludis*	Bardy and Jarrell (2002); Hu et al. (2011)
**Signal peptide peptidases (SPP)**				Degrades signal peptides after removal by SP		
SppA (TK1164)	S49	Active site at the soluble extracytoplasmic side	1 TMS	First insight into catalytic mechanism of prokaryotic SPPs	*Thermococcus kodakaraensis*	Matsumi et al. (2005, 2006)
**Presenilin/SPP homolog**	A22	Active site at the membrane plane (ICliP)		Regulatory intramembrane proteolysis (RIP). Aspartyl proteases (GxGD)		
PSH; MCMJR1 peptidase			9 TMS	Crystal structure determined in an archaeon. Insights on catalytic mechanism of Presenilin and SPP intramembrane proteases	*Methanoculleus marisnigri*	Li et al. (2013)
**ATP-dependent proteases**				Protein quality control. Regulatory proteases		
**LonB**	S16	ATPase and protease domains facing cytoplasm				
(TK1264)			2 TMS	ATP-independent activity on unfolded substrates; ATP-dependent activity on folded proteins	*T. kodakaraensis*	Fukui et al. (2002)
(Ta1081)			2 TMS	Confirmation of S-K catalytic dyad. Recombinant LonB protease showed ATPase and protease activities	*Thermoplasma acidophilium*	Besche and Zwickl (2004); Besche et al. (2004)
(AF0364)			1 TMS	Crystal structure of LonB proteolytic domain solved	*Archaeoglobus fulgidus*	Botos et al. (2005)
(TON_0529)			2 TMS	Crystal structure 2.0- resolution solved	*Thermococcus onnurineus*	Cha et al. (2010)
(Nmag_2822)			2 TMS	DNA-binding capacity *in vitro*. Can complement a LonB mutant in *H. volcanii* suggesting functional conservation of LonB	*Natrialba magadii*	Sastre et al. (2011); Cerletti et al. (2014)
(HVO_0783)			2 TMS	Essential for viability. Quality control of proteins. Suboptimal LonB expression affects the content of membrane carotenoids and other lipids	*H. volcanii*	Cerletti et al. (2014)
**Rhomboids**	S54	Active site at the membrane plane (ICliP)		Regulatory intramembrane proteolysis (RIP)		
Rho II (HVO_ 0727)			6 TMS	A null mutant in *rhoII* gene showed reduced mobility and higher sensitivity to novobiocin. The mutation affected N-glycosylation of the S-layer glycoprotein with a sulfoquinovose containing oligosaccharide	*H. volcanii*	Parente et al. (2014)
**Site 2 proteases (S2P)**	M50B	Active site at the membrane plane (ICliP)		Regulatory intramembrane proteolysis (RIP)		
(MJ_0392)			6 TMS	The crystal structure of the transmembrane core was determined (3B4R)	*M. jannaschii*	Feng et al. (2007)
**Archaeosortases**	N/A	Predicted active-site Cys oriented toward the extracellular side				
(HVO_0915)			7 TMS	Involved in C-terminal processing of the S-layer glycoprotein	*H. volcanii*	Abdul Halim et al. (2013)
**Thermopsine** (SSO2194)	A5	Active site oriented to the extracellular milieu	1 TMS	Overproduced in peptide enriched media. Catalytic domain followed by PKD and Y_Y_Y domains	*S. solfataricus*	Cannio et al. (2010)
**CAAX prenyl proteases**	M79			In eukaryotic cells they are involved in prenylation of proteins for membrane localization		
Abi (HVO_0784)			5 TMS	Putative CAAX protease homolog. Forms a transcription unit with *lonb* gene. A null mutant showed no evident phenotypes	*H. volcanii*	Cerletti et al. (2014)

## MEMBRANE-ASSOCIATED PROTEASES OF *ARCHAEA*

An overview of the repertoire of membrane proteases that occur in archaeal cells is shown in Table S1 based on *in silico* examination of the complete genome sequences of some representative archaea members. Some protease families are widely represented among archaeal genomes such as HtpX homologs, LonB, SP, and Site 2 proteases (S2Ps) whereas others are restricted to a limited number of organisms (for instance the protease families A5, M10, and PrsW protease). **Table 1** describes the membrane proteases that have been experimentally characterized from the *Archaea* domain. Some of them have been studied in more detail (SPI and TFPP-like SP) and at least a few of their endogenous substrates have been identified (e.g., preflagellins, prepilins, and sugar-binding proteins for TFPP-like peptidases). However, most families have been examined to a limited extent or remain uncharacterized, and their biological relevance and/or targets are unknown (e.g., rhomboids, LonB, CAAX prenyl protease homologs, S2Ps).

The crystal structures of a number of archaeal membrane proteases have been solved (*Methanococcus maripaludis* FlaK; *Thermococcus onnurineus* and *Archaeoglobus fulgidus* LonB proteolytic domains; S2P transmembrane segments (TMSs) core from *Methanococcus jannaschii;* MCMJR1 peptidase from *Methanoculleus marisnigri*) providing valuable structure/function insights on these protease families (see **Table 1** for references).

## MEMBRANE PROTEASES IMPLICATED IN THE ASSEMBLY OF THE ARCHAEAL CELL ENVELOPE AND SURFACE STRUCTURES

Probably one of the most distinctive features of archaea is their ability to survive in environments with extremely adverse conditions that are lethal for most life forms. To this end, they have adapted their physiology and cellular structures. One such instance is the cell envelope. The archaeal cell envelope is composed of an atypical cellular membrane constituted by isoprenyl ether glycerol phospholipids surrounded by surface S-layer proteins as the major (or sole) component of the cell wall (Albers and Meyer, 2011). These structures maintain the cellular integrity and functionality as well as serve as a shell to cope with the harsh conditions predominating in their surroundings (Claus et al., 2005).

In addition to the S-layer, archaea show very diverse and complex cell surface structures (reviewed in Lassak et al., 2012). The biogenesis of the appendages composed of bacterial type IV pilin subunits, the pili and the archaeal flagellum or archaellum, has been characterized to some extent. These structures play important roles in cell motility as well as in surface attachment, DNA exchange and cell-cell interaction.

**FIGURE 1:**
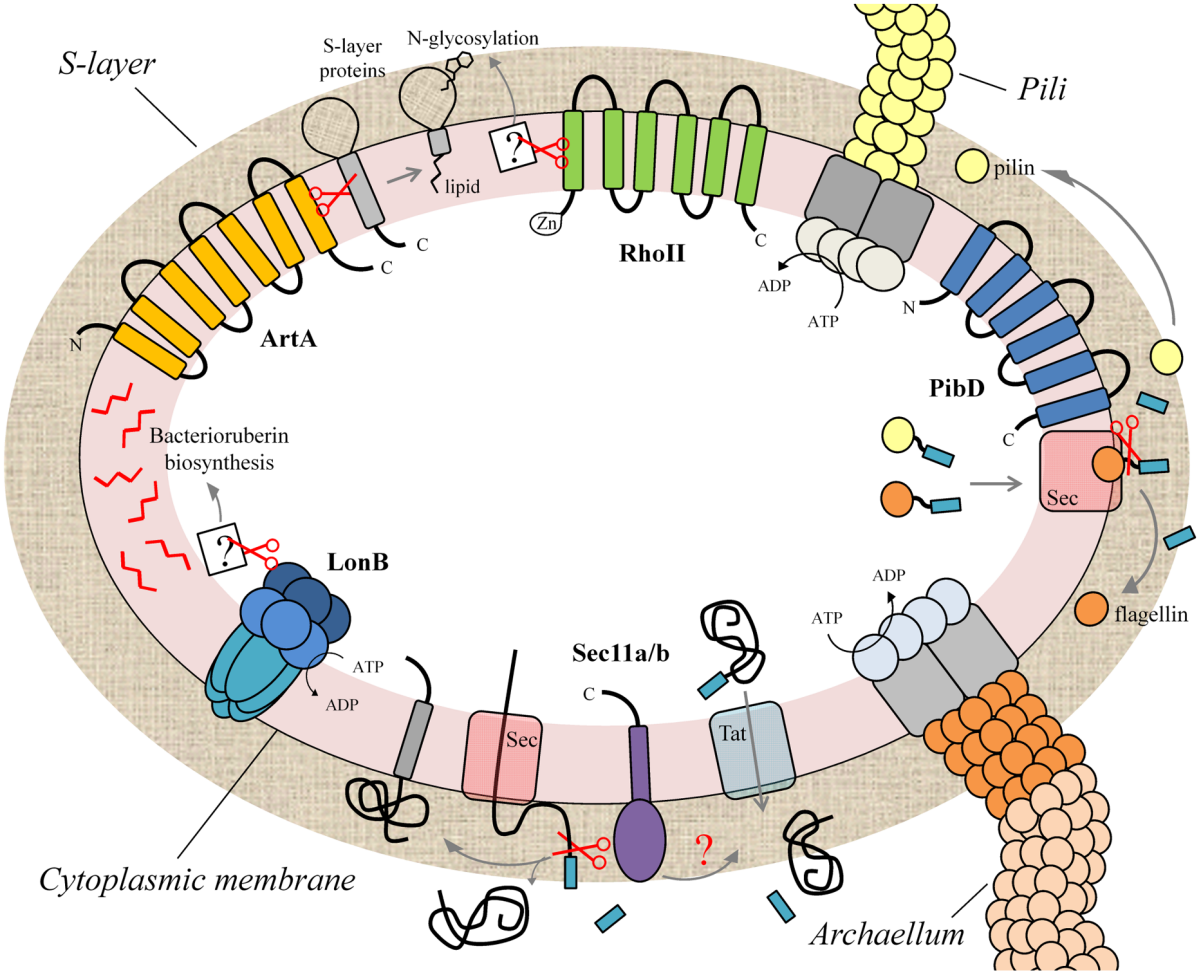
**Membrane proteases implicated in the biogenesis of the cell envelope and extracellular appendages of the haloarchaeon *Haloferax volcanii*.** Schematic representation of different protease families that participate in processes leading to the biogenesis of the cell envelope (cytoplasmic membrane, S-layer) and cell surface structures (archaellum, pili) in *H. volcanii,* and probably in other archaea. LonB is implicated in bacterioruberin biosynthesis and protein quality control. The signal peptidases Sec11a/b process the signal peptide of secreted and membrane proteins translocated through the Sec pathway. PibD cleaves preflagellins and prepilins, the protein components of the archaellum and pili. The rhomboid protease RhoII is involved in the *N*-glycosylation of the S-layer protein with a sulfoquinovose-containing oligosaccharide while archaeosortase ArtA mediates the proteolytic processing coupled-lipid modification of this glycoprotein facilitating its attachment to the archaeal cell surface.

Haloarchaea, a very diverse and probably the best characterized group of archaea, flourish in habitats with high salinity (> 2M NaCl) and intense solar irradiation. In the haloarchaeon *H. volcanii* the structure and maturation of the S-layer glycoprotein as well as the biogenesis of pili and flagella have been examined (Jarrell et al., 2010; Kaminski et al., 2013; Kandiba et al., 2013; Tripepi et al., 2013; Esquivel and Pohlschroder, 2014). The adequate localization and functionality of these structures requires the participation of different families of proteases which are immersed in the context of the cytoplasmic membrane. Below we describe the recent advances on the membrane-associated proteases involved in the processes leading to the assembly of the cell envelope and surface structures in the euryarchaeon *H. volcanii*. The currently available information is summarized in **Figure 1**.

### PROTEASES INVOLVED IN THE BIOGENESIS OF THE CYTOPLASMIC MEMBRANE AND SECRETION OF PREPROTEINS

The quality control of membrane proteins is essential for proper cell physiology. In bacteria and eukaryotic organelles a major role in this process is performed by the energy-dependent membrane protease FtsH (Dalbey et al., 2012; Langklotz et al., 2012). Archaea possess only two ATP-dependent proteases: the 20S proteasome (soluble enzyme) and an unusually membrane-bound version of the Lon protease (LonB). The archaeal LonB probably resembles functionally to the FtsH protease which is absent in archaea. LonB has been biochemically and/or structurally characterized in several archaeal members (**Table 1**). In agreement with the genomic prediction, LonB has been immunolocalized in association with the cell membrane in the haloarchaea *Natrialba magadii* and *H. volcanii*. The recombinant protease derived from *N. magadii* (NmLon) showed DNA binding capacity *in vitro*, a feature in common with LonA proteases (Sastre et al., 2011). As FtsH is for *Escherichia coli* (Langklotz et al., 2012), LonB is essential for viability of *H. volcanii* cells. On the other hand suboptimal expression of this protease affects growth rate, cell shape, antibiotic sensitivity, and lipid composition (Cerletti et al., 2014). Also, *H. volcanii* mutant cells deficient in Lon content are more sensitive to puromycin compared to wild type cells suggesting that LonB is involved in the disposal of abnormal proteins. A distinctive feature of haloarchaea is the presence of red membrane-bound carotenoid pigments (C50-bacterioruberins) which serve to protect their macromolecules from the damaging effects of UV light (Khanafari et al., 2010). Interestingly, the cellular content of bacterioruberins dramatically increased in *H. volcanii* mutant cells with a suboptimal Lon concentration while overexpression of this protease rendered the cells colorless (Cerletti et al., 2014). This observation suggests that LonB controls carotenoid biosynthesis in *H. volcanii* probably by degrading enzyme/s involved in this pathway. It is likely that deregulation of the cellular concentration of bacterioruberins and other lipids affects membrane stability contributing to the lethal phenotype of the *lon* knock out mutant.

Signal peptidases are central in the protein secretion process as they remove signal peptides from secretory and membrane-bound polypeptides. In archaea, type I signal peptidase (SPI), type IV prepilin peptidase (TFPP)-like enzymes and signal peptide peptidase (SPP) have been characterized. A detailed description on the distribution and properties of these enzymes has been previously reported (Ng et al., 2007). SPIs process the majority of pre-proteins that are translocated through the general secretion pathway (Sec), however, whether this enzyme also cleaves Tat signal peptides remains to be demonstrated. Like all members of the SPI family, archaeal SPIs are serine proteases and based on studies performed in SPI from *M. voltae* (Ng and Jarrell, 2003) and *H. volcanii* (Fink-Lavi and Eichler, 2008) the catalytic mechanism of the archaeal SPI homolog seems to rely on a Ser/His/Asp tryad resembling the eukaryotic enzyme. In *H. volcanii* two different SPIs with distinct efficiency for substrate cleavage exist, Sec11a and Sec11b, however, only Sec11b is essential for viability (Fine et al., 2006). It is likely that these enzymes exert different roles and/or cleave distinct substrates *in vivo*.

SPII removes signal peptides from lipoproteins. Although there are numerous proteins in archaea that contain signal peptides with the lipobox motif, including several predicted to be secreted via the Tat pathway, homologs of bacterial SPII have not been identified in archaeal genomes (Giménez et al., 2007). Thus, it has been proposed that a distinct enzyme may exist in archaea to process prelipoproteins (Ng et al., 2007).

### PROTEASES INVOLVED IN MATURATION OF THE CELL WALL (S-LAYER GLYCOPROTEIN)

In *H. volcanii* the S-layer glycoprotein is the sole structure that constitutes the cell wall. This protein has been used to examine the molecular/structural adaptations of haloarchaeal proteins to high salt and has served as a model to study protein glycosylation in archaea (Eichler et al., 2013; Jarrell et al., 2014). In haloarchaea, maturation of the S-layer glycoprotein requires at least three different types of posttranslational modifications: glycosylation, proteolytic cleavage and isoprenylation (Konrad and Eichler, 2002; Eichler, 2003). The glycosylation process of the S-layer has been recently reviewed (Eichler et al., 2013).

Sortases are cysteine proteases from Gram-positive bacteria that “sort” proteins to the cell surface by covalently joining them to the cell wall or polymerize pilins to build pili (Proft and Baker, 2009; Clancy et al., 2010; Hendrickx et al., 2011; Spirig et al., 2011). These enzymes modify surface proteins by recognizing and cleaving a sorting signal located either in the N or *C*-terminus of the target protein. Many genomes in bacteria and archaea encode proteins containing a *C*-terminal domain with structural similarity to the *C*-terminus of sortase substrates. These proteins coexist in these genomes with at least one member of the protease families denoted as exosortases (bacteria) or archaeosortases (archaea). Exo and archaeosortases are polytopic membrane proteins with no sequence homology to bacterial sortases. However, they contain the conserved cysteine, arginine, and histidine residues found in the active site of sortases suggesting that they may perform similar functions (Haft et al., 2012). Recently it was reported that *H. volcanii* mutant cells with a deletion in the archaeosortase gene *artA* showed growth defects (which were more evident under low-salt conditions), alterations in cell shape and the S-layer organization, impaired motility and decreased conjugation rates (Abdul Halim et al., 2013). This work demonstrated that ArtA is involved in *C*-terminal processing of the S-layer glycoprotein suggesting that archaeosortases are functional homologs of bacterial sortases. Considering the location of the archaeosortase recognition sequence (PGF) immediately following the TMS of the substrate protein, it was proposed that this enzyme may facilitate the covalent attachment of target proteins (e.g., S-layer glycoprotein) to a membrane lipid in contrast to sortases which attach proteins to the growing cell wall.

Rhomboids are membrane serine proteases involved in regulatory intramembrane proteolysis (RIP) and are conserved in the three domains of life (Lemberg, 2013). The catalytic mechanism of rhomboids relies on a Ser/His dyad located in different TMS of the protease to cleave membrane protein substrates. In eukaryotic cells the functions of this protease family are very diverse and include cell-cell signaling, development, apoptosis, organelle integrity and parasite invasion (reviewed in Freeman, 2014). The relevance of rhomboids in the prokaryotic cell physiology is scarcely understood. In bacteria, rhomboid null mutants show phenotypes that may be related to defective cell envelope and/or cell-surface structures. In *Bacillus subtilis*, a mutant strain in the rhomboid homolog YqgP displayed a slight decrease in glucose uptake and a defect in cell division leading to the formation of filamentous cells (Mesak et al., 2004); *Mycobacterium smegmatis* rhomboid mutants showed reduced capacity for biofilm formation and increased sensitivity to antibiotics (Kateete et al., 2012). So far only TatA, a protein component of the Tat translocon in the pathogenic bacterium *Providencia stuartii,* has been experimentally confirmed as a rhomboid substrate (Stevenson et al., 2007). In this organism the rhomboid protease AarA cleaves an *N*-terminal extension of TatA which in turn allows for secretion of an unknown quorum sensing signal. Archaea appear to encode various sequences for rhomboid proteases (Table S1). In haloarchaea, homologs with various topologies can be found including proteins with six or more TMS as well as unusual rhomboids containing an AN-1 Zn-finger domain at the *N*-terminus. *H. volcanii* has two putative genes for rhomboids, RhoI (nine TMS) and RhoII (six TMS, with *N*-terminal AN-1 Zn finger domain). A knock-out mutant of *rhoII* in *H. volcanii* displayed mild defects in motility and novobiocin sensitivity. This mutant strain was also affected in the glycosylation of the S-layer. In *H. volcanii* wild type cells the S-layer glycoprotein Asn732 is bound to an oligosaccharide containing at least 6 repeating units of sulfoquinovose-hexose (SQ-Hex) while in the mutant strain this residue contained only two SQ-Hex suggesting that RhoII controls (directly or indirectly) the protein glycosylation process in *H. volcanii* (Parente et al., 2014).

### PROTEASES INVOLVED IN THE BIOGENESIS OF CELL SURFACE APPENDAGES

In bacteria, the precursors of type IV pilins and related pseudopilins are processed by a special enzyme belonging to a novel aspartic acid protease family, the type IV prepilin signal peptidase (SPIV/TFPP; Ng et al., 2009). In contrast to SPI and SPII, this enzyme cleaves the signal peptides directly after the *n*-region leaving the *h*-region bound to the mature protein facilitating anchoring/assembly of pilin subunits onto the cell surface (Ng et al., 2007). Archaea encode TFPP-like proteins and they have been studied with regard to their role in the assembly of the structures composing the motility apparatus (**Table 1**). The archaellum is composed of unique proteins that are unrelated to bacterial flagellins. Archaeal preflagellins contain short signal peptides at the *N*-terminus which are similar to those of bacterial type IV pilins, the protein components of pili. These filamentous surface structures facilitate twitching motility in bacteria. TFPP-like proteases process the signal peptides of archaeal preflagellins. The enzymes present in *M. maripaludis* and *Methanococcus voltae* (FlaK), *Sulfolobus solfataricus,* and *H. volcanii* (PibD) are the most extensively characterized TFPPs of archaea (see references in **Table 1**). FlaK and PibD show some divergences including the length of the signal peptide, key amino acid residues surrounding the cleavage site as well as substrate preference (Ng et al., 2009). PibD from *S. solfataricus* and *H. volcanii* has a broader substrate selection than FlaK, as, in addition to preflagellins, these enzymes can mature prepilins (Albers et al., 2003; Tripepi et al., 2010). *S. solfataricus* PibD also processes certain sugar-binding proteins of the “bindosome,” filamentous-like structures that extend from the cell surface (Albers et al., 2003; Szabo et al., 2006).

The *H. volcanii* genome encodes flagellins and contains genes for other type IV pilin-like proteins. Tripepi et al. (2010) showed that deletion of *pibD* disrupted preflagellins processing and prevented maturation of type IV pilin-like proteins. The mutant cells were non-motile and were unable to adhere to a glass surface. These results suggest that PibD is needed for maturation of preflagellins and other type IV pilin-like proteins in *H. volcanii*.

Recently, based on *in vivo* analysis of the catalytic activity of *Sulfolobus acidocaldarius* PibD, TFPPs were renamed as GxHyD group of proteases (rather than DxGD; Henche et al., 2014).

## CONCLUDING REMARKS

In prokaryotes the assembly and composition of cell surface structures are essential for the adjustment to the varying conditions of the environment and to interact with their surroundings (e.g., establish cell-cell and/or cell-substrate contacts). In the haloarchaeon *H. volcanii*, several membrane-associated proteases are implicated in different processes (protein secretion, processing and sorting) leading to the biogenesis of the cell wall and extracellular appendages (**Figure 1**), highlighting the importance of these enzymes in the adaptation and interaction of archaea with their environment.

Structural analysis of archaeal membrane proteases (Flak and GxGD proteases) have advanced the knowledge on the catalytic and molecular mechanism of intramembrane cleaving proteases. This will help to understand the mechanism of the eukaryotic homologous enzymes which are implicated in human physiology (regulation of immune response) and/or in the development of diseases (e g. Alzheimer).

There are still many open questions in this field: e.g., endogenous substrates of most membrane proteases are unknown. Efforts should continue to better understand the role of membrane proteases in archaeal physiology.

## AUTHOR CONTRIBUTIONS

All authors made substantial contributions to the acquisition, analysis and interpretation of data for this review. All authors critically reviewed and edited the manuscript, and approved the final version before submission to publication. All authors agree to be accountable for all aspects of the work in ensuring that questions related to the accuracy or integrity of any part of the work are appropriately investigated and resolved.

## Conflict of Interest Statement

The authors declare that the research was conducted in the absence of any commercial or financial relationships that could be construed as a potential conflict of interest.
