# Supplementary material for: Archaeal membrane-associated proteases: insights on Haloferax volcanii and other haloarchaea
Source: Front Microbiol. 2015 Feb 6;6:39. doi: 10.3389/fmicb.2015.00039 (PMC4343526; doi:10.3389/fmicb.2015.00039)
Supplement: Supplementary file 1 [file table_s1.doc]

| **Locus tag** | **Protein name** | **TMS** | **MEROPS Family** | **Distribution*** | **Reference** |
| --- | --- | --- | --- | --- | --- |
| ***Membrane-associated proteases*** | | | | | |
| HVO_0915, HAH_1990, Nmag_2570, MJ1469.1, MM_1401, MM_2114, VNG2669G | Archaeosortase | 3-9 | N/A | N/A | Abdul Halim et al., 2013 |
| SS01886, SS02037, SS2045, SS02194 | Thermopsine/SsMTP | 1 | A5 | 21 | Cannio et al., 2010 |
| HVO_2993, MM_1678, SS00131, TK0053 | Prepilin peptidase (pibD) | 6 | A24 | 57 | Albers et al., 2003; Szabo et al., 2006; Ng et al., 2009; Tripepi et al., 2010; Henche et al., 2014 |
| MJ0835.2, mvol_1220, mvol_0489 | EppA protease | 8 | A24A | 13 | Szabo et al., 2007 |
| HHA_2955, MJ0902, MJ1282.1, [VNG2285C](http://halolex.mpg.de/public/getdetails.jsf?strain=Halobacterium_sp.NRC-1.public_NCBI&orfid=VNG2285C), RMVO00262, mvol_0164 | FlaK-domain containing protein/Preflagelin peptidase | 2-5 | A24B | 12 | Bardy and Jarrell, 2002 and 2003; Ng et al., 2009; Hu et al., 2011 |
| MM_0919 | Unassigned peptidase | 1 | M10A | 17 |  |
| HVO_0102, HVO_2904, HHA_0174, HHA_0757, HHA_1943, HHA_5268, Nmag_0968, Nmag_1286, Nmag_1859, Nmag_2035, Nmag_2505, Nmag_2745, Nmag_2766, Nmag_2952, Nmag_3113, MJ1682, MM_1236, MM_3134, MM_2115, PF1135, PF1597, SS0323, SS01859, SS02694, VNG0129G, [VNG0329G](http://halolex.mpg.de/public/getdetails.jsf?strain=Halobacterium_sp.NRC-1.public_NCBI&orfid=VNG0329G), VNG0408H, mvol_1656, TK2258, TK0677 | HtpX protease | 2-7 | M48 | 118 |  |
| HVO_0784, Hvo_1997, Nmag_2572, Nmag_2712, Nmag_4079, MM_2190, VNG0354C, VNG1993H | CAAX protease/Abortive infection protein (Abi) | 3-11 | M79 | 33 | Cerletti et al., 2014 |
| HVO_0408, HAH_2807, Nmag_1405, PF2049, VNG2625C, TK1387 | PrsW peptidase | 7-9 | M82 | 34 |  |
| MJ1512 | Unassigned peptide lyase | 1 | N10 | 43 |  |
| MM_1373, TK1401 | Unassigned peptidase | 1 | S9C | 76 |  |
| Nmag_2602, Nmag_4139 | Beta lactamase | 4 | S12 | 31 |  |
| HVO_0783, HAH_2425, Nmag_2822, MJ1417, MJ1318, MM_0128, MM_1913, PF0467, TK1264, PF1438, VNG0303G, mvol_0998, TK1264, TK2168 | ATP-dependent Lon protease | 1-2 | S16 | 109 | Fukui et al., 2002; Besche et al., 2004; Im et al., 2004; Botos et al., 2005; Cha et al., 2010; Sastre et al., 2011; Cerletti et al., 2014 |
| HVO_2603, HVO_0002, HAH_0243, Nmag_1326, Nmag_3375, Nmag_3743, MJ0260, MM_1344, PF0326, VNG2416G, mvol_1688, TK1703, TK2037 | Signal peptidase | 1-3 | S26 | 103 | Ng and Jarrell, 2003; Fine et al., 2006; Fink-Lavi and Eichler, 2008 |
| Nmag_2775, MM_3122 | Alpha/beta hydrolase fold protein | 1 | S33 | 83 |  |
| HVO_0881, Nmag_2612, Nmag_2635, MJ0651, PF1583, VNG_0303G, VNG_0620G, mvol_0155, mvol_1409, TK1164 | Proteinase IV like/Archaean signal peptide peptidase II | 1-2 | S49A | 31 | Matsumi et al, 2005; Matsumi et al., 2006 |
| MM_0866 | Periplasmic serine protease | 1 | S49B | 22 |  |
| MJ0137, MM_2032, PF0240, PF1533, TK00347 | Archaean signal peptide peptidase 1 | 1-4 | S49C | 54 |  |
| ***Intramembrane cleaving proteases*** | | | | | |
| HVO_1107, HAH_0799, Nmag_3602, MM_0909, VNG1365C | Presenilin homolog/MCMJR1 peptidase | 7-9 | A22.B | 51 | Li et al., 2013 |
| HVO_0285, HVO_1862, HVO_1870, HHA_0041, HHA_0440, HHA_2180, HHA_2714, Nmag_1508, Nmag_1514, Nmag_2136, MJ0392, MJ0611, MJ0971, MM_1318, MM_1523, MM_3009, PF0167, PF0392, PF0457, SS0008, VNG0875Cm, VNG2019C, VNG2168C, VNG2012C, mvol_1182, TK1820, TK1247 | SpoIVFB-type metallopeptidase/S2P family metalloprotease/Putative membrane-associated Zn-metallo protease/MjS2P-type peptidase | 3-9 | M50 | 112 | Feng et al., 2007 |
| HVO_0727, HVO_1474, HHA_1328, HHA_3058, HHA_1218, Nmag_1128, Nmag_1636, Nmag_2518, Nmag_3579, Nmag_4170, MJ0610.1, MM_0682, PF1228, SSo0463, VNG0361C, VNG0858C, TK0786 | Rhomboid protease | 6-10 | S54 | 70 | Parente et al., 2014 |

* Archaea containing at least one homolog out of 146 archaeal genomes available at the MEROPS database. Organisms abbreviations: HHA: *Haloarcula hispanica*; VNG: *Halobacterium sp* NRC-1; HVO: *Haloferax volcanii*; MJ: *Methanococcus jannaschii*; mvol (or RMVO): *Methanococcus voltae* MM: *Methanosarcina mazei*; Nmag: *Natrialba magadii*; PF: *Pyrococcus furiosus*; Sso: *Sulfolobus solfataricus*; TK: *Thermococcus kodakaraensis*

N/A: Not available in MEROPS Database
